# Closing the Yield Gap of Sugar Beet in the Netherlands—A Joint Effort

**DOI:** 10.3389/fpls.2018.00184

**Published:** 2018-02-22

**Authors:** Bram Hanse, Frans G. J. Tijink, Jurgen Maassen, Noud van Swaaij

**Affiliations:** IRS (Institute of Sugar Beet Research), Dinteloord, Netherlands

**Keywords:** sugar beet, yield potential, grower's management, pests and diseases, soil structure, harvest losses, agronomy, extension

## Abstract

The reform of the European Union's sugar regime caused potential decreasing beet prices. Therefore, the Speeding Up Sugar Yield (SUSY) project was initiated. At the start, a 3 × 15 target was formulated: in 2015 the national average sugar yield in the Netherlands equals 15 t/ha (60% of the sugar beet potential) and the total variable costs 15 euro/t sugar beet, aspiring a saving on total variable costs and a strong increase in sugar yield. Based on their average sugar yield in 2000-2004, 26 pairs of “type top” (high yielding) and “type average” (average yielding) growers were selected from all sugar beet growing regions in the Netherlands. On the fields of those farmers, all measures of sugar beet cultivation were investigated, including cost calculation and recording phytopathological, agronomical and soil characteristics in 2006 and 2007. Although there was no significant difference in total variable costs, the “type top” growers yielded significantly 20% more sugar in each year compared to the “type average” growers. Therefore, the most profitable strategy for the growers is maximizing sugar yield and optimizing costs. The difference in sugar yield between growers could be explained by pests and diseases (50%), weed control (30%), soil structure (25%) and sowing date (14%), all interacting with each other. The SUSY-project revealed the effect of the grower's management on sugar yield. As a follow up for the SUSY-project, a growers' guide “Suikerbietsignalen” was published, Best Practice study groups of growers were formed and trainings and workshops were given and field days organized. Further, the benchmarking and feedback on the crop management recordings and the extension on variety choice, sowing performance, foliar fungi control and harvest losses were intensified. On the research part, a resistance breaking strain of the Beet Necrotic Yellow Vein Virus (BNYVV) and a new foliar fungus, *Stemphylium beticola*, were identified and options for control were tested, and implemented in growers practices. The joint efforts of sugar industry, sugar beet research and growers resulted in a raise in sugar yield from 10.6 t/ha in 2002-2006 to 13.8 t/ha in 2012-2016.

## Introduction

Historically, the share in farmers income from the sugar beet crop was relatively high (Berkhout and Berkum, 2005). In those years, the sugar regime of the European Union (EU) guaranteed minimum sugar beet prices for quota beet and cause a relative stable income compared to other crops of which the prices are fluctuating within and between years, like carrots, onions and potatoes (Berkhout and Bruchem, 2005, 2010; Vrolijk et al., 2009). With the sugar market reform of 2006 the European Union lowered the guaranteed sugar beet price for farmers from 43.63 euro/t sugar beet (EC, 2001; Zeddies, 2006) to 26.29 euro/t from 2009 onwards (EC, 2006), which is decrease of a 39.7%. This causes a dramatic drop in farmers' income when the costs remain on a similar level. After the sugar marketing year 2016/2017 the system of sugar quotas is abolished (EP and EC. Regulation 1308, 2013) with a high price volatility in a free market as an expected result.

A study on the inputs of sugar beet production in the Netherlands called Low Input Sustainable Sugar Yield (LISSY), identified possibilities to save up to 20% of the total variable costs (Pauwels, 2006). To keep the profitability of the sugar beet crop on the level of before 2006, an increase in yield is needed because the savings on the total variable costs could not compensate the sugar beet price drop. Early research estimated the potential sugar yield in the Netherlands at 23 t sugar/ha (De Wit, 1953), while the average sugar yield realized by growers in the period 2002–2006 was 10.6 t/ha only 46% of the estimated potential (Van Swaaij, 2007). Large differences in yield levels between growers in the same region, with the same production circumstances like soil and climate, are reported frequently (Agrarische Dienst, 2007). This phenomenon is not restricted to sugar beet production in the Netherlands, it's found in Sweden (the 4T project), Germany and the United Kingdom (Blomquist et al., 2003; Fuchs et al., 2008; Limb and Atkin, 2010). Also for other crops large differences in yield levels among growers are reported as well (Lobell et al., 2009). Although large differences exist, it seems that in many cases the average yield of other crops is close to 80% of the crops potential in that region (Lobell et al., 2009). This unexploited yield gap in sugar beet cultivation and the possibilities of high price volatility in future, was the reason for the IRS (Institute of Sugar Beet Research, The Netherlands) to initiate a chain of research and knowledge transfer in Dutch sugar beet production. The basic idea was that stable high yields at farm level is the best strategy to compensate for high price volatility. This chain approach included research (SUSY-project) and knowledge exchange by extension via Best Practice Groups, field days and trainings of harvester drivers, crop specialists and crop advisors. At the start of the SUSY-project a 3 × 15 target was formulated: in 2015 the national average sugar yield in the Netherlands equals 15 t/ha (60% of the sugar beet potential) and the total variable costs 15 euro/t sugar beet, aspiring a saving on total variable costs and a strong increase in sugar yield.

## Materials and methods

### SUSY-project

The SUSY-project (Speeding Up Sugar Yield) studied the difference in sugar yield of growers in a pairwise comparison (Hanse, 2011). Growers were selected based on their sugar yields in the period 2000–2004. A grower with high yields (“type top”) and a grower with average yields (“type average”) which were neighbors formed a pair in the study. Both growers of a pair encountered the same production prerequisites: soil and climate. The pairwise comparison comprised 26 pairs (52 growers). A “type top” grower was defined as a grower with sugar yields in the period 2000-2004, on average and in each single year above the 75% quartile of the region where the farm was situated. A “type average” grower was defined as a grower with sugar yields among the 50% quartile in that region in the same period. Within a formed pair, the yield level of a “type top” and a “type average” grower differed at least 1.5 ton sugar per hectare based on the 5 years average between those two growers (Hanse, 2011).

From the participating growers data on parameters of soil physics, soil fertility, soil health, rainfall, drilling (date, depth, distance), field establishment, canopy closure, pests and diseases, nutrient uptake, yield, and quality, harvest losses and exact field size (GPS) were collected in 2006 and 2007. Next to that, the growers recorded all agronomic measurements, including application dates, prices, type and amounts of consumables etc. In 2008 only the exact field size, harvest losses and yield data was recorded next to the agronomic measurements. The SUSY-study, the measurements, recorded data and statistical methods are described in more detail in Hanse et al. (2010, 2011a,b). The yields of the participating growers from 2016 were taken and compared with their yields of 2006 without correction for harvest losses. The obtained data were analyzed using GenStat, 18th edition (VSN International Ltd.). To analyse the effect of grower, location and their interactions, linear mixed models were used. The pair rank number, region and the interaction of both, were used as random terms (random model) to analyse the “type top” and “type average” growers within a pair directly with each other.

### Best practice groups

In the period 2007–2010, 37 Best Practice groups were formed. The groups consisted of 13 farmers on average (smallest group 9 and biggest 18) which followed a voluntary 2-years' program under the supervision of a crop specialist of the Agricultural Department of the sugar industry. The crop specialists were trained for the supervision of the Best Practice groups. The aim of the Best Practice groups was to exchange and deepen knowledge and experience between the participating farmers. Five meetings were held annually and after the first meeting the topics for a year were selected, which were prepared by a subgroup of three to four group members. Meetings were held on the farm of one of the participating farmers with a field visit to discuss on sugar beet growing. At the end of the 2 years' period each Best Practice Group formulated tips for sugar beet growers. The first results of the SUSY-project and the first Best Practice group meetings among daily practice of the crop specialists of Suiker Unie brought up the idea to produce a practical guide Sugar Beet Signals (In 't Hout and Maassen, 2008).

### Field days

From the results generated by the SUSY-project and discussed in the Best Practice groups, topics for field days were selected. Fifteen field days were organized from 2007 onwards on locations within sugar beet growing areas, one field day per year moving from area to area in subsequent years, finally covering the whole of the Netherlands. The field days were organized with a guided tour for the visitors. A tour had multiple topics explained by an expert in circa 10–15 min with 5 min for questions from the audience and lasted in total for 1 to 2 h.

### Integrated management of foliar fungi

To raise the awareness of sugar beet growers of foliar fungi and how to recognize them, the project “Integrated management of foliar fungi in sugar beet” was initiated (fund of the Ministry of Agriculture of the Netherlands). The goal of this project (2006-March 2008) was communication and knowledge transfer on foliar fungi in sugar beet. At the end of the project the impact was monitored by a telephone inquiry before the start of the project (autumn 2005) and after the project (spring 2008).

### Harvester driver training and workshops diagnostics of pests and diseases

The results of the SUSY-project gave also rise to the idea to train harvester drivers with the aim to minimize harvest losses. In 2009 and 2011 a harvester driver training was held. The training lasted for 1 day with an introduction on harvest and harvest quality of sugar beets and minimizing harvest losses. In the afternoon the group of drivers divided in subgroups based on the brand of harvester they were working with daily. Technicians of each manufacturer, which also participated in the introduction, explained for their machine the possible adjustments for adapting the machine to the circumstances in the field and minimizing harvest losses. The effect of making adjustments was real time tested on an available sugar beet field for each brand of machine separately.

The importance of pests and diseases on sugar yield found in the SUSY-project (Hanse et al., 2011a) initiated a series of workshops on the recognition or diagnosis of pests and diseases. The aim was to increase the ability of crop specialists and crop advisors to recognize pests and diseases in sugar beet. Workshops were typically setup with a short introduction on the importance of the right diagnosis of pests and diseases for all participants, following with the task to diagnose 20–40 randomized samples obtained from the IRS Diagnostic Service (Raaijmakers et al., 2014). During the provided time of 1 h IRS diagnostic specialists helped the participants with pointing at symptoms and showing out the subtle difference, without directly diagnose the concerning sample. After 1 h, the answers were provided and questions on the samples were answered centrally. Also more detailed information for the management of the pests and diseases concerning were provided. In 2012 and 2013 one workshop with the topic “Recognition of foliar fungi” was held in Bergen op Zoom (at the IRS facilities) and Valthermond (at the facilities of the local research farm of Wageningen University & Research), respectively. A workshop with the topic “Early season diagnostics” was held in Bergen op Zoom (2014) and Valthermond (2015). A workshop with the topic “Late season diagnostics” was held in Rolde (at the facilities of the local research farm of Wageningen University & Research; 2014) and Bergen op Zoom (2015).

### Development of sugar yield in the Netherlands

The average sugar yields in the Netherlands of 1950–2016 were analyzed with non-linear and split-line regressions to estimate the effect of the total chain approach on sugar yield and identify the breakpoint in time. For the regression analyses, the statistical package GenStat, 18th edition (VSN International Ltd.) was used. The effect of breeding on sugar yield level for the period 2006–2016 was analyzed using the variety choice and the yield data of the official variety trials in the Netherlands using the same methodology as described by Rijk et al. (2013).

## Results

### SUSY-project

The sugar yields of “type top” growers were significantly 20% higher in comparison to the “type average” growers, but the total variable costs did not differ significantly between both grower types (Table 1). The sugar yield differences between growers were explained by pests and diseases (50%), weed control (30%), soil structure (25%), and sowing date (14%), all interacting with each other (Hanse, 2011). Within the category of pests and diseases on the clay soils *Heterodera schachttii* and BNYVV infestation levels were found to be important variates explaining sugar yield levels, and on sandy soils the number of fungicide applications, *Aphonomyces cochlioides* and *Heterodera betae* infestation levels (Hanse et al., 2011a). Harvest losses were initially recorded to correct the sugar factory delivered yield into grown yield on a participating field. They were found surprisingly high during the project. Total harvest losses (whole beet losses, losses due to root tip breakage and too deep topping), were on average 2.9 t/ha, minimum 0.45 t/ha and maximum 9.1 t/ha (Hanse and Tijink, 2010). Those variates became topics of further research and extension in the Netherlands, especially in harvester driver training. The sugar yields without harvest losses of the participants of the SUSY-project, 10 years after the project are shown in Table 2. The significant difference in sugar yield level between “type top” and “type average” growers disappeared in the 10 years after the start of the project. Although the average sugar yield is 1 t/ha higher for the “type average” in 2016, this difference is not significant (*P* = 0.586). This is due to the large variation in the 2016 yield data caused by extreme rainfall in early summer (June and July) in the South East causing low yield or even crop failures. With regard to the national yield level, both “type top” and “type average” are yielding at the 75% quartile level in 2016. Table 2 also shows the national sugar yield level for the average and 75% quartile. The national yield level of 2016 is 22% higher compared to the yield level of 2006. On the national level, the difference between the 50% quartile and 75% quartile in 2006 and 2016 is comparable (12.8 and 13.1%, respectively).

**Table 1:** Influence of grower type on yield and costs in Dutch sugar beet production; SUSY-project, 2006–2008.

**Grower**	**Root yield (t/ha)^a^**	**Sugar content (%)**	**Sugar yield (t/ha)^a^**	**Revenues (euro/ha)**	**Total variable costs (euro/ha)^b^**
type top	78.1	17.21	13.4	3,099	1,416
type average	66.7	17.01	11.4	2,618	1,356
LSD 5%	2.89	0.22	0.51	128.8	73.35
P	≤0.001	≤0.05	≤0.001	≤0.001	n.s.

*Data from Hanse et al. (2010)*.

a *Yield not corrected for harvest losses*.

b *Costs mentioned exclude the fixed costs e.g. tenancy for the field and the overhead of the farm. The overhead encloses profit margin, costs of sugar quota, assurances for crop and grower, buildings, maintenance of fields, field and ditch edges*.

*n.s. means not significant*.

**Table 2:** Sugar yield in 2006 and 2016 of “type top” and “type average” growers participating the SUSY-project in the Netherlands.

**Grower**	**SUSY-project**				**The Netherlands**	
	**Number**		**Sugar yield (t/ha)**		**Sugar yield (t/ha)**	
	**2006**	**2016**	**2006**	**2016**	**2006**	**2016**
“type top”	26	23	12.8	15.2	12.5	15.3
“type average”	26	22	10.9	16.2	10.9	13.3
LSD 5%			0.80	6.99		
P			<0.001	0.586		

### Best practice groups

Almost 500 growers participated in the Best Practice study groups. At the end of 2010, when the last started Best Practice groups finished the first years' period, the tips formulated by each Best Practice group were listed to 15 tips in total and printed on the back side of each paper of a block note. Block notes were distributed to each sugar beet grower visiting the regional winter meetings or study groups (Table 3).

**Table 3:** Fifteen tips for a high sugar yield from sugar beet growers participating the Best Practice groups.

**Number**	**Tip**
1	grow your sugar beet conscious for the highest profit, review critically every handling and watch how colleagues are doing it
2	have a wide as possible crop rotation and take care for the right soil pH
3	use an acreage as low as possible to fulfill contract obligations
4	beet cyst nematode tolerant varieties pays back quickly, already from a low infestation level
5	cherish your soil, the reward is a high yield
6	when the soil is dry enough, sow as soon as possible
7	conduct soil treatments preferably in a single pass
8	choose the lowest tire pressure from the table; low tire pressure saves soil structure, fuel and time
9	fertilization with nitrogen, phosphorus and potassium can often be more economical
10	ask a colleague grower why he is doing things, listen to his arguments, don't judge too quickly and try to get benefit out of it for yourself
11	be keen on weeds and spray on seedlings, prevent hardening of weeds
12	be alert for foliar fungi and perform the first fungicide application on time (first infection at that field)
13	harvest what is grown, pay attention to top, tip and whole beet losses; topping 1 mm to deep means 1% of nett root loss!
14	store beets dry, cool and frost free. A fleece cover will keep your beets dry
15	stay informed on what is going on and register for the free e-mail service of IRS (www.irs.nl)

### Field days

Fifteen field days were organized across the sugar beet growing area's in the Netherlands (Table 4). On average 414 sugar beet growers visited the field days, implying that in each region large numbers of sugar beet growers got informed by the topics identified in het SUSY-project and the Best Practice groups.

**Table 4:** Field days on sugar beet growing organized in the Netherlands (2007–2017).

**Year**	**Location**	**Demonstration**	**Topics in guided tour**	**Growers**
2007 (October)	Colijnsplaat (southwest NL)	- harvest and topping - tyre pressure and fuel consumption	- beet cyst nematode management - verticillium wilt - green manure crops - diagnostics of pests and diseases - soil management - control of foliar fungi	400
2008 (October)	Valthermond (northeast NL)	- harvest and topping - tyre pressure and fuel consumption	- beet cyst nematode management - control of foliar fungi - fertilization - yellow spots (*Stemphylium beticola*)	550
2009 (June)	Valthermond (northeast NL)	- volunteer potato control - mechanical weed control	- soil treatments and seed bed preparation - optimal Nitrogen rate - cleaning spraying equipment - chemical weed control - variety choice	500
2009 (September)	Vredepeel (southeast NL)	tyre pressure and fuel consumption	- harvest and topping - control of foliar fungi - sugar beet as energy crop - fertilization and water quality - soil management - maize for biogas - trichodorid nematodes - rhizoctonia tolerant variety choice	450
2010 (October)	Lelystad (central NL)	Beet Europe 2010; demonstration of 10 sugar beet harvesters by manufacturers with independent test 2 days before	- storage after harvest - variety choice - green manure crops - nitrogen application rate - control of foliar fungi - effect of worn out drilling disks on crop uniformity	1,200
2011 (June)	Munnekezijl (north NL)	spray technique (drift reduction)	- variety choice - nitrogen application techniques - manganese fertilization - effect of worn out drilling disks on crop uniformity - symptoms of herbicide damage	400
2011 (September)	Wijnandsrade (south NL)	- tyre pressure - soil compaction - soil treatment	- spray technique - storage after harvest - beet cyst nematode management - soil profile	350
2013 (June)	Valthermond (northeast NL)	none	- variety choice - leaf miner control - control of *Stemphylium beticola*	200
2014 (August)	Valthermond (northeast NL)	none	- optimal soil pH for sugar beet in a rotation with a high share (33–50%) of potatoes - soil treatment - variety choice - control of *Stemphylium beticola*	250
2015 (February)	Dronten (central NL)	precision sowing machines	- effect of grower on emergence and uniform crop stand - seedbed preparation - soil treatment and adjustment of equipment - GPS usage	180
2015 (June)	Valthermond (northeast NL)	none	- variety choice - control of *Stemphylium beticola* - optimal soil pH for sugar beet in a rotation with a high share of potatoes (33-50%) - diagnostics of pests and diseases	200
2015 (July)	Vredepeel (southeast NL)	spray technique (drift reduction)	- increasing the humus content in the soil - mechanical weed control - diagnostics of pest and diseases - control of foliar fungi - rhizoctonia tolerant variety choice - nitrogen and phosphorus application	300
2016 (June)	Lelystad (central NL)	tyre pressure and soil compaction	- long term phosphorus application - liquid fertilizers - weed control with 75% drift reduction nozzles - spray application and drift reduction - diagnosis of pests and diseases - variety choice	350
2016 (September)	Wijnandsrade (south NL)	harvest quality	harvest quality	140
2017 (August)	Westmaas (southwest NL)	none	- harvest quality - soil treatment and soil structure - liquid fertilizers - green manure crops - tyre pressure at 100 kPa - drones for crop inspection - diagnosis of pests and diseases - variety choice	750

### Integrated management of foliar fungi

Within this project an interactive map to visualize the regional warnings when foliar fungi were found in the different sugar beet growing regions, was developed and made online accessible. This interactive map was visited 8,712 times from 1 October 2005 till 17 March 2008. To improve the recognition of the foliar fungi in sugar beet and provide information on the fungi species and their management, a special website was developed in which the interactive map was incorporated as well. In the period from 1 October 2005 till 17 March 2008 this website received 13,042 visits. The website and interactive map remains online and are accessible via www.irs.nl/bladschimmel. The inquiries before and after the project revealed that sugar beet growers became more aware of the foliar fungi. In 2005, 42% of the growers applied fungicides against foliar fungi and in 2007 79% of the growers. The increased attention of foliar fungi management in the extension resulted in more attention of growers for this topic. Also the recognition of foliar fungi and timing of applications was improved after the project (Table 5).

**Table 5:** Results extension project “Integrated management of foliar fungi” (2006-2008).

**Activity**	**Number**	**Growers reached**	**Remarks**
regional winter meetings	66	8,500	management of foliar fungi topic in program
regional demostration strips	13	1,100	
publications in growers magazine	10	All (14,000)	
internet articles	32	9,800 visits via e-mail notifications	inspired 45 articles in agricultural press

### Harvester driver training and workshops diagnostics of pests and diseases

At the harvester training day of 2009, 30 drivers participated and in 2011, 40. This training has since 2012 a follow up with Harvest Checks by the crop specialists of the sugar industry. At the workshop for the recognition of foliar fungi, 55 crop specialists and crop advisors participated from the south of the Netherlands. In 2013, the same workshop had 40 participants in the north. The workshop in early season diagnostics had 58 participants in Bergen op Zoom (2014) and 37 in Valthermond (2015). The late season diagnostics workshop had 49 participants in Rolde (2014) and 60 in Bergen op Zoom (2015).

### Development of sugar yield in the Netherlands

**Figure 1:**
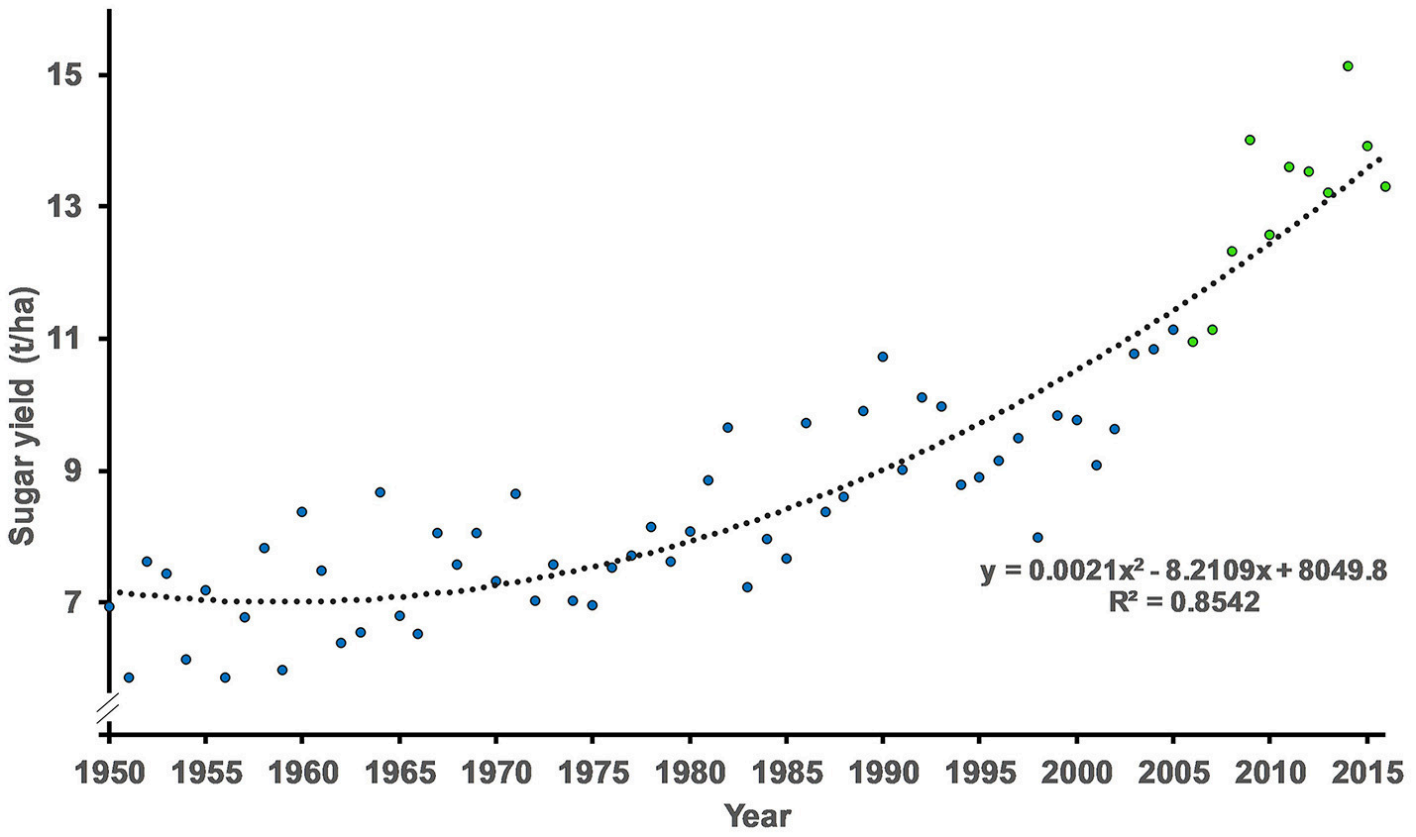
The average sugar yield in the Netherland (1950–2016). The period 1950–2005 is indicated in blue and had a yearly sugar yield increase of 0.9%. The period 2006–2016 is indicated in green and had a sugar yield increase of 3.4% a year.

The average sugar yield in the Netherlands from 1950 to 2016 is shown in Figure 1. The split-line regression identified a break in the trend after the year 2000. In the first period from 1950 till the breakpoint the yearly sugar yield increase was 0.06 t/year (0.9%) and in the period after the breakpoint 0.33 t/year (3.4%). The effect of breeding was estimated as 1.0% in the period 2006-2016. In that period the use of resistant varieties as a tool to circumvent damage by pests and diseases increased. In 2006 the share of rhizomania resistant varieties in the Netherlands was 97%, in 2016 100%. The share of rhizomania resistant varieties with rhizoctonia tolerance increased from 17% in 2006 to 26% in 2016. Also the share of rhizomania resistant varieties with beet cyst nematode tolerance increased from 2% in 2006 to 41% in 2016. Next to that in 2016, 1.2% of the acreage was grown with a rhizomania resistant variety which combines the rhizoctonia and beet cyst nematode tolerance (triple resistance). In 2016, 17% of the rhizomania resistant varieties had two major resistance genes (*Rz*1 + *Rz*2). In 2006 this two last categories of varieties were not available on the national variety list.

## Discussion

To keep the sugar beet crop profitable in the Netherlands, the sugar yield level is extremely important. The SUSY-study showed that there was no relation between total variable costs and sugar yield level. The conclusion was drawn that the most profitable strategy for sugar beet growers, preparing for future uncertainties in a market with high price volatility, is the maximizing sugar yield with simultaneously optimizing costs (Hanse et al., 2010). The SUSY-project also found a huge impact of the grower on sugar yield level. Also other studies underlined the importance of the grower's management for the sugar yield level (Fuchs et al., 2008; Trimpler et al., 2017). The effect a grower has on the sugar yield level was the motivation to organize the Best Practice groups, field days, the project on foliar fungi, the trainings of harvester drivers and the workshops on diagnostics of pests and diseases. The central topics for the field days arose or were underlined from the results of the SUSY-project. An example of a topic that arose from the SUSY-project are the harvest losses initially intended to measure in order to correct the sugar factory delivered yield to field grown yield. Already after the first year of the SUSY-project it showed up as a factor with a quick win and potential to improve due to the measured variance among growers on similar soil types (Hanse and Tijink, 2010). The management of foliar diseases is an example of a topic underlined by the SUSY-project. The importance for sugar beet production was addressed just before the start of the SUSY-project by Vereijssen et al. (2007) with the project on integrated management of foliar fungi as a follow up. That latest, extension based, project resulted in almost a doubling of growers applying fungicides to protect their sugar beets from foliar fungi (increase of 42 to 79% in 2 years' time). However, the yellow leaf spots appearing from 2007 onwards caused new research on foliar diseases resulting in the identification of *Stemphylium beticola*, a new foliar fungus in sugar beet (Hanse et al., 2015; Crous et al., 2016; Woudenberg et al., 2017). The management of *S. beticola* became an important topic at field days and the recognition and diagnosis at the workshops on diagnosis of pests and diseases. These workshops were also organized for the diagnosis of most common pests and diseases in sugar beet growing by crop advisors and crop experts, since pests and diseases explained a large part of the difference in sugar yield of “type top” and “type average” growers in the SUSY-project. Despite crop protection measures applied the participating growers lost 24% of their sugar yield to pests and diseases (Hanse et al., 2011a). This result is quite similar to the estimated losses to pest and diseases in sugar beet worldwide (Oerke and Dehne, 2004). Therefore, also new research (and subsequent extension) was generated on the management of *Heterodera betae* (Raaijmakers, 2014). This nematode species was known to be present, but the SUSY-project pointed out the impact on sugar yield on sandy soils, urging for options of control. At the first sight, the impact of rhizomania on sugar yield levels on clay soils was curious, since the whole sugar beet acreage was sown with rhizomania resistant varieties from 2007 onwards. Further investigation showed that on fields with rhizomania symptoms in a *Rz*1 resistant variety a resistant breaking P25 tetrad (AYPR) of the Beet Necrotic Yellow Vein Virus (BNYVV) A-type occurred (Bornemann et al., 2015). The spread of this tetrad type caused an increase in the share of *Rz*1*Rz*2 rhizomania resistant varieties. Finally, the results in the SUSY-project on the white beet cyst nematode, *Heterodera schachtii*, caused more extension on the choice of the right variety, with a shift to a share of 41% nematode tolerant varieties in 2016. The annual increase in sugar yield showed a clear discontinue trend and raised from 0.9 to 3.4% after the breakpoint. One explanation of the yield increase in sugar beet is the genetic improvement by breeding. Studies on the breeding progress estimate a 0.7–2.0% yearly increase of sugar yield based on variety trials (Scott and Jaggard, 2000; Zimmermann and Zeddies, 2000; Märländer et al., 2003; Koch, 2006). In field research with stored seeds which were tested under equal agronomical and climatological conditions a breeding progress of 0.9% was found (Loel et al., 2014), while different resistance traits against pathogens were not included (Loel et al., 2014). The resistance against pathogens is an essential part of the breeding progress (Jansen and Stibbe, 2007). Compared with potatoes and cereals, having a linear yield increase, the yield increase of sugar beet is convex, showing a larger effect in yield increase than breeding progress could explain (Rijk et al., 2013). Analysis of the yield gap of sugar beet producing countries showed that the Netherlands had the highest increase in sugar yield (Jaggard et al., 2012). This study also suggest an effect of agronomy (or management) in the sugar yield increase, while the breeding effort for all countries is similar. It also revealed that progress in yield in variety trials and in practise developed parallel in the Netherlands. Despite changes in weather growers in the Netherlands were able to achieve the same speed of progress in yield increase; in most other countries the yield gap between variety trials and delivered beet was increasing. Analysis of the variety choice and the yield data of the official variety trials in the period 2006–2016 in the Netherlands showed that breeding was responsible for a 1.0% average yield increase per year. The remaining increase in sugar yield is mainly due to the management of the grower, interacting with the weather conditions encountering on his fields. The effect of climate change on sugar yield level in the Netherlands is unclear. Positive effects on sugar beet yield might be reduced by negative effects, resulting in a very small or even zero effect (Van Oort et al., 2012). A crop model simulation by Reidsma et al. (2015) found substantial effects of climate change (increasing temperature and annual rainfall) on sugar beet yields. However, the factor management was set to zero for sugar beets in this study. The analyses by Rijk et al. (2013) could not disentangle environment and management. There might also be an influence of grower's management on the impact of climate change on crop yield, for instance: “type top” growers had a higher rooting depth and potentially suffer less from the longer periods of drought and had a better soil structure below plowing depth as well, giving the field more capacity in case of excessive rainfall (Hanse et al., 2011b). The development of the sugar yield in the Netherlands shows a clear discontinue trend. This is due to the effect growers can have on yield once they make the right choice on the right time before and during the season. The whole integrated extension effort described in this manuscript supported the growers in their management. The effect of the grower is once more underlined by the results of the “type average” growers in the SUSY-project, 10 years after the project the difference in yield level with the “type top” growers is vanished, thus raising the average yield level. This could be due to more attention to the crop and solving some of the management issues by the “type average” growers. The difference of the 50 and 75% quartile of the national sugar yield of 12.8 and 13.1% in 2006 and 2016 respectively, indicates that there is still potential left among all sugar beet growers in the Netherlands for a further future yield increase.

## Author contributions

There was an equal contribution of the authors to this manuscript. All authors participated in discussing the focus, data to select, analyse and present this data in tables, describing results and putting it to discussion.

### Conflict of interest statement

The authors declare that the research was conducted in the absence of any commercial or financial relationships that could be construed as a potential conflict of interest.
